# Total fertilization failure and idiopathic subfertility

**DOI:** 10.1186/1477-7827-7-3

**Published:** 2009-01-12

**Authors:** Sandra J Tanahatoe, Joseph McDonnell, Angelique J Goverde, Peter GA Hompes, Cornelis B Lambalk

**Affiliations:** 1 Department of Obstetrics, Gynaecology and Reproductive Medicine, Vrije Universiteit medical centre, Amsterdam, The Netherlands; 2 Department of Obstetrics, Gynaecology and Reproductive Medicine, Vrije Universiteit medical centre, PO Box 7057, 1007 MB Amsterdam, The Netherlands; 3 Department of reproductive Medicine and Gynaecology, University Medical Centre Utrecht, The Netherlands

## Abstract

**Background:**

To gain more insight in whether failure of intrauterine insemination (IUI) treatment in patients with idiopathic subfertility could be related to diminished fertilization, the aim of this study is to compare the fertilization of an initial IVF procedure after six cycles of IUI and the fertilization of an initial IVF procedure without preceding IUI cycles in couples with idiopathic subfertility.

**Methods:**

We performed a complimentary analysis of a randomized controlled trial, in which the number of total fertilization failure (TFF) in the first IVF procedure after unsuccessful IUI was compared to those of IVF without preceding IUI in patients with idiopathic subfertility. These patients participated in a previous study that assessed the cost effectiveness of IUI versus IVF in idiopathic subfertility and were randomized to either IUI or IVF treatment.

**Results:**

45 patients underwent IVF after 6 cycles of unsuccessful IUI and 58 patients underwent IVF immediately without preceding IUI. In 7 patients the IVF treatment was cancelled before ovum pick. In the IVF after unsuccessful IUI group TFF was seen in 2 of the 39 patients (5%) versus 7 of the 56 patients (13%) in the immediate IVF group. After correction for confounding factors the TFF rate was not significantly different between the two groups (p = 0.08, OR 7.4; 95% CI: 0.5–14.9).

**Conclusion:**

Our data showed that TFF and the fertilization rate in the first IVF treatment were not significantly different between couples with idiopathic subfertility undergoing IVF after failure of IUI versus those couples undergoing IVF immediately without prior IUI treatment. Apparently, impaired fertilization does not play a significant role in the success rate of IUI in patients with idiopathic subfertility.

## Background

It is generally accepted that patients with long term idiopathic subfertility should be treated with intrauterine insemination (IUI), because it is shown to be the most cost effective treatment [1]. However, a considerable number of patients have not conceived after 6 cycles of IUI. The question remains why these couples do not conceive with IUI treatment. IUI is thought to increase pregnancy chances by enhancing the exposure of one or more female gametes to a large number of male gametes. However, it is possible that factors exist that impair fertilization, implantation and placentation that cannot be identified by extensive subfertility investigation.

It is hypothesized that impaired fertilization could be a result of oocyte or sperm dysfunction [2-4]. If impaired fertilization is one of the causes of subfertility, it could play a role in failure of IUI treatment and could result in fertilization failure of an IVF procedure in these patients. From this point of view, fertilization in vitro could be the ultimate test of sperm and/or oocyte function.

Gurgan et al showed in a retrospective case control study that patients with idiopathic subfertility undergoing IVF-ET after 4–6 unsuccessful IUI cycles showed significantly more total fertilization failure (TFF) events compared to patients undergoing IVF-ET because of tubal factor subfertility [5]. The incidence of TFF was 20.4% and 7.6% in the idiopathic and tubal factor group respectively (*P *< 0.005). Moreover, TFF tended to be repetitive in couples with idiopathic subfertility. This higher incidence of TFF events in couples with idiopathic subfertility may be attributed to elimination of couples with more fertilizable gametes in previous IUI cycles.

It has been shown that fertilization rates are lower in idiopathic subfertility compared to tubal factor subfertility [4,6,7]. This appears to confirm the hypothesis that a fertilization factor could be an underlying cause of idiopathic subfertility. On the other hand, in couples with idiopathic subfertility directly undergoing IVF instead of IUI treatment, TFF does not necessarily persist during subsequent IVF cycles [8]. Whether fertilization failure in idiopathic subfertility plays a role in unsuccessful IUI, is unknown.

If a fertilization disorder is an undiscovered cause of unsuccessful IUI treatment in idiopathic subfertility, then this could be revealed in a subsequent IVF treatment by the occurrence of TFF or low fertilization rate. If this is the case then it could be useful to detect this factor in order to prevent unnecessary and costly treatment with IUI and/or IVF. Theoretically, aside from being a therapeutic procedure an IVF attempt could be a diagnostic test in vitro for fertilization, provided that the fertilization process in vitro reflects the same situation in vivo. If fertilization is present a couple can start IUI treatment. In case of TFF it could be more cost effective to change treatment into ICSI after an IVF attempt has resulted in total fertilization failure.

To gain more insight in whether failure of IUI treatment in patients with idiopathic subfertility could be related to fertilization failure, the aim of this study is to compare the number of TFF of an initial IVF procedure after six cycles of IUI with an initial IVF procedure without preceding IUI cycles in couples with idiopathic subfertility.

## Methods

### Patients

We performed a complimentary analysis of a randomized controlled trial in which the occurrence of TFF in the first IVF procedure after unsuccessful IUI was compared to that of IVF without preceding IUI in patients with idiopathic subfertility. Patients with idiopathic subfertility who underwent IVF after 6 unsuccessful IUI cycles were compared to patients with idiopathic subfertility who underwent IVF immediately without preceding IUI treatment. These patients participated in a previous study that assessed the cost effectiveness of IUI versus [1] IVF in idiopathic and male subfertility. In that study, patients were randomized to either IUI or IVF treatment after they had given their informed consent. Those patients who underwent 6 cycles of IUI without a positive pregnancy test in the trial were offered IVF as standard patient care after they had left the trial.

For this study approval from the institute's review board was acquired. Couples affected by male subfertility were excluded to avoid bias of impaired semen quality on fertilization rate and TFF. Male subfertility was diagnosed if at least three out of five semen analyses showed a total motile sperm count of fewer than 20 million progressively motile spermatozoa in the ejaculate, and if the remainder of the subfertility investigation showed no additional abnormalities. Couples had been diagnosed as having idiopathic subfertility if no abnormality is found after full subfertility investigation and duration of subfertility of at least 3 years. Subfertility investigation consisted of a basal body temperature chart, a post coital test, a late luteal phase endometrial biopsy, a hysterosalpingography, a diagnostic laparoscopy and at least two semen samples.

### IVF Procedure

The IVF procedure in all patients was carried out as described by Goverde et al [1]. Women aged 38 years or younger underwent controlled ovarian hyperstimulation with a "long" protocol with gonadotropin-releasing-hormone agonist (Decapeptyl, Ferring, Copenhagen, Denmark). Gonadotropins were given in a daily dose of two to three ampoules (150–225 IU) of human menopausal gonadotropin (Pergonal, Ares Serono) or follicle-stimulating hormone, depending on the patient's age or previous response to gonadotropins. In women older than 38 years, a "short" stimulation protocol was applied. In both protocols, gonadotropin-releasing-hormone agonists and gonadotropins were discontinued if transvaginal ultrasonography showed the presence of at least one follicle with a diameter of at least 18 mm, and a minimum of three follicles of at least 16 mm in diameter. 36 h before follicle aspiration, 10 000 IU human chorionic gonadotropin was given unless the serum oestradiol concentration exceeded 20 000 nmol/L. Follicular aspiration guided by transvaginal ultrasonography was done under systemic analgesia (7·5 mg diazepam orally and 50 mg pethidine hydrochloride intramuscularly), and all follicles present were aspirated. The retrieved oocytes were cultured in Earls'+ medium (Sigma, St Louis, MO, USA), and inseminated with Percoll-processed spermatozoa 42 h after the human chorionic gonadotropin injection. We transferred a maximum of two pre-embryos in woman 35 years of age or younger, and of three pre-embryos in women older than 35 years, 48–72 h after oocyte retrieval.

The luteal phase was supported by three doses of progesterone (200 mg; Progestan, Nourypharma, Oss, Netherlands) intravaginally daily from the day of oocyte retrieval, or, in the case of breakthrough bleeding before the 13th day of the luteal phase under progesterone treatment, by 1500 IU human chorionic gonadotropin (Pregnyl, Organon, Oss, Netherlands) intramuscularly every 48 h, starting from the second day after oocyte retrieval until a pregnancy test was done at the 15th day after oocyte retrieval.

To prepare semen, fresh and liquefied ejaculates were processed over a Percoll gradient (40/90 gradient) by centrifugation at 750 g for 15 min. The pellet was resuspended in 2 mL Earls'+ medium. The isolated spermatozoa were spun down at 300 g for 7 min, and this pellet was resuspended in 2 mL of culture medium and stored in 5% carbon dioxide in air at room temperature. Just before insemination, the spermatozoa were spun down at 200 g for 7 min.

Insemination took place between 38 and 42 hours after hCG. Eighteen hours after the moment of insemination, the fertilization of the oocytes were estimated in terms of the number of pronuclei; oocytes with two or more pronuclei were included as fertilized.

Directly before the transfer procedure, the embryo development and morphology score were determined and the best embryos were selected. Each embryo was scored 1 to 4 according to its symmetry and the extend of fragmentation. Embryo transfer was generally executed on day 3 after oocyte retrieval. If only two or fewer embryos were available the transfer was performed on day 2 after oocyte retrieval.

### Statistical analysis

The primary endpoint of the study was the incidence of TFF and secondary the mean fertilization rate of the first IVF procedure in both patient groups. TFF is defined as absence of fertilization of all inseminated oocytes. Fertilization rate was calculated as the number of embryos divided by the number of inseminated oocytes. To assess whether the groups were comparable, baseline characteristics were listed, such as age, primary or secondary subfertility, duration of subfertility and total progressively motile sperm count (TPMC) after processing.

Comparison of TFF and fertilization rate were calculated after correction of factors that could influence fertilization, such as age and TPMC after processing and differences in stimulation if any. For univariate analysis the student's *t *test was used to analyze continuous data and the χ^2 ^test for discrete data. For multivariate analysis logistic or linear regression analysis were used.

## Results

### Patients

In the initial study by Goverde et al. a total of 181 couples with idiopathic subfertility were randomized to either IUI or IVF (1). Of these, 120 patients were assigned to IUI and 61 to IVF. Of the patients who underwent IUI treatment first, 45 couples continued with IVF after unsuccessful IUI treatment, 36 patients became pregnant due to IUI, 39 patients stopped treatment before, during or after IUI treatment. Of the 61 patients assigned to IVF 58 actually obtained treatment and 3 patients withdrew their informed consent. As shown in table 1 baseline characteristics were comparable in both groups regarding age, duration of subfertility and primary subfertility (table 1).

**Table 1:** Baseline characteristics

	IVF after unsuccessful IUI (n = 45)	IVF immediately (n = 58)	p-value
Female age in years	33.3 (3.9)	32.6 (3.7)	0.32
Duration of subfertility in years	5.2 (2.0)	4.7 (2.0)	0.22
Primary subfertility (%)	33 (73%)	45 (78%)	0.62
Secondary subfertility (%)	12 (27%)	13 (22%)	

Values are mean ± standard deviation (SD) unless indicated otherwise

IVF = in vitro fertilization, IUI = intrauterine insemination

During the stimulation phase 8 patients dropped out (table 2). These included 6 patients in the IVF after unsuccessful IUI group, five because of stimulation failure and one because of hyperstimulation. In this patient oocyte retrieval and in vitro fertilization was performed but embryo transfer did not take place. Two patients in the IVF immediately group dropped out because of stimulation failure so follicle aspiration was cancelled in these patients. One patient in the IVF after unsuccessful IUI group did not have any oocytes retrieved at follicle aspiration.

**Table 2:** Treatment characteristics

	IVF after unsuccessful IUI (n = 45)	IVF immediately (n = 58)	p-value
Number of canceled cycles	6 (13%)^a^	2 (3%)^b^	0.15
Total number of follicles^c^	11.1 (7.6)	11.7 (6.0)	0.67
Total number of oocytes retrieved	9.2 (6.3)	9.7 (5.9)	0.66
Total progressively motile sperm count (×10^6^)^d^	21.3 (21.7)	26.3 (22.8)	0.28
Total number of embryos	6.4 (4.6)	5.5 (3.9)	0.35
Fertilization rate	68 (27.7)	63 (34.7)	0.48
Number of TFF	2/39 (5%)	7/56 (13%)	0.23
Positive pregnancy test (hCG>5IU/l)	11 (25%)	12 (21%)	0.64
Clinical pregnancy rate	8 (18%)	11 (19%)	0.88
Ongoing pregnancy rate	6 (13%)	9 (16%)	0.76

Values are mean ± standard deviation unless indicated otherwise

^a ^5 patients cancelled due to absence of ovarian response. 1 patient cancelled due to hyperstimulation

^b ^both cancelled due to absence of ovarian response

^c ^follicles > 10 mm on day of hCG administration

^d ^after processing of semen samples used for insemination

IVF = in vitro fertilization, IUI = intrauterine insemination, TFF = total fertilization failure

### Treatment outcome

Oocytes were retrieved and fertilized in 39 patients in the IVF after unsuccessful IUI group versus 56 in the group starting IVF immediately. The mean number of follicles, oocytes retrieved, embryos obtained and good quality embryos were not significantly different between groups (table 2). Surprisingly, total fertilization failure showed no difference between the groups, 2 out of 39 (5%) patients in the IVF after unsuccessful IUI group versus 7 out of 56 (13%) patients in the group starting IVF immediately (p = 0.23, OR 0.38; 95% confidence interval (CI): 0.07–1.92). Even after adjustment for age and TPMC after processing TFF showed no difference between groups (p = 0.08, OR 7.4, 95% CI 0.5–14.9). The mean fertilization rate too was not significantly different between groups, 67.8% in the IVF after unsuccessful IUI group versus 63% in the group starting IVF immediately (p = 0.48). Also after adjustment for age and TPMC after processing the fertilization rate between both groups was not different with a p-value of 0.38.

A positive pregnancy test was seen in 11 (25%) and in 12 (21%) patients in respectively the IVF after unsuccessful IUI group and the group starting IVF immediately (p = 0.64, OR 0.80; 95% CI: 0.31–2.03). There was no difference in clinical pregnancy rate or in ongoing pregnancy rate (table 2). In the IVF after unsuccessful IUI group 6 (13%) ongoing pregnancies were seen versus 9 (16%) in the other group (p = 0.76, OR 0.84; 95% CI: 0.27–2.55).

## Discussion

The nature of this study was to asses whether failure of IUI treatment in patients with idiopathic subfertility could be related to fertilization failure. Our data, however, showed that the incidence of TFF and the mean fertilization rate in the first IVF treatment were not significantly different between couples with idiopathic subfertility undergoing IVF after failure of IUI versus those couples undergoing IVF immediately without prior IUI treatment.

Although several studies showed lower fertilization rates and more often TFF in idiopathic subfertility compared to tubal subfertility [4-7], our data suggest that the contribution of impaired fertilization to negative IUI outcome seems to be limited. On the contrary, the number of TFF was surprisingly high in those patients receiving IVF immediately compared to those receiving IVF after unsuccessful IUI treatment (13% respectively 5%), but not significantly different.

Whether TFF in the first IVF cycle actually reflects a fertilization disorder in vivo is a matter of debate. TFF is observed in 5–20% in couples with normal sperm count undergoing IVF, with a substantial recurrence rate of 30–67% [9-12]. In a prospective study comparing the efficacy of IVF and ICSI after TFF in a previous IVF attempt, the fertilization rate in a second cycle was significantly higher after ICSI than after IVF (48% respectively 11%, P < 0.001). The TFF recurrence rate in this study was 67% in the second cycle of IVF [12]. This high recurrence rate of TFF implies that TFF in the first cycle could reflect a fertilization disorder.

Whether such a fertilization disorder also leads to unsuccessful IUI outcome is still a question. Because TFF tends to be repetitive in a high number of patients and that TFF is more common in patients with idiopathic subfertility after 4–6 unsuccessful IUI cycles [5], it is possible that unsuccessful IUI in idiopathic subfertility is associated with a fertilization disorder. Therefore, we hypothesized that the incidence of TFF would be higher in patients with idiopathic subfertility, who failed to become pregnant after IUI compared with those who underwent IVF immediately. If this would be the case in a substantial number of patients, it could be clinically relevant to find out if a diagnostic IVF procedure would prevent unnecessary treatment with IUI. In the contrary, we could not find a difference in the incidence of TFF between the groups. Apparently, if there is a fertilization factor, it does not play a significant role in the success rate of IUI in patients with idiopathic subfertility. Further prospective studies should be performed to investigate the role of fertilization failure and unsuccessful IUI in idiopathic subfertility.

## Conclusion

Our data showed that there is no evident relation between failure of IUI treatment and fertilization failure in the first IVF cycle. Therefore, performing a so called diagnostic IVF procedure to assess a possible fertilization factor would not contribute to a better and/or more cost-effective treatment strategy for couples with idiopathic subfertility and IUI remains the first treatment of choice.

## Competing interests

The authors declare that they have no competing interests.

## Authors' contributions

SJT participated in the design of the study and drafted the manuscript. JM carried out the statistical analysis. AJG participated in the design of the study and carried out the collection of the participating couples. CBL and PGAH participated in the design of the study. All authors read and approved the manuscript.

## References

[B1] Goverde AJ, McDonnell J, Vermeiden JP, Schats R, Rutten FF, Schoemaker J (2000). Intrauterine insemination or in-vitro fertilisation in idiopathic subfertility and male subfertility: a randomised trial and cost- effectiveness analysis. Lancet.

[B2] Hull MG, Williams JA, Ray B, McLaughlin EA, Akande VA, Ford WC (1998). The contribution of subtle oocyte or sperm dysfunction affecting fertilization in endometriosis-associated or unexplained infertility: a controlled comparison with tubal infertility and use of donor spermatozoa. Hum Reprod.

[B3] Wolf JP, Bulwa S, Ducot B, Rodrigues D, Jouannet P (1996). Fertilizing ability of sperm with unexplained in vitro fertilization failures, as assessed by the zona-free hamster egg penetration assay: its prognostic value for sperm-oolemma interaction. Fertil Steril.

[B4] Audibert F, Hedon B, Arnal F, Humeau C, Badoc E, Virenque V, Boulot P, Mares P, Laffargue F, Viala JL (1989). Results of IVF attempts in patients with unexplained infertility. Hum Reprod.

[B5] Gurgan T, Urman B, Yarali H, Kisnisci HA (1995). The results of in vitro fertilization-embryo transfer in couples with unexplained infertility failing to conceive with superovulation and intrauterine insemination. Fertil Steril.

[B6] Mackenna AI, Zegers-Hochschild F, Fernandez EO, Fabres CV, Huidobro CA, Prado JA, Roblero LS, Altieri EL, Guadarrama AR, Lopez TH (1992). Fertilization rate in couples with unexplained infertility. Hum Reprod.

[B7] Navot D, Muasher SJ, Oehninger S, Liu HC, Veeck LL, Kreiner D, Rosenwaks Z (1988). The value of in vitro fertilization for the treatment of unexplained infertility. Fertil Steril.

[B8] Lipitz S, Rabinovici J, Ben Shlomo I, Bider D, Ben Rafael Z, Mashiach S, Dor J (1993). Complete failure of fertilization in couples with unexplained infertility: implications for subsequent in vitro fertilization cycles. Fertil Steril.

[B9] Roest J, van Heusden AM, Zeilmaker GH, Verhoeff A (1998). Treatment policy after poor fertilization in the first IVF cycle. J Assist Reprod Genet.

[B10] Molloy D, Harrison K, Breen T, Hennessey J (1991). The predictive value of idiopathic failure to fertilize on the first in vitro fertilization attempt. Fertil Steril.

[B11] Barlow P, Englert Y, Puissant F, Lejeune B, Delvigne A, Van Rysselberge M, Leroy F (1990). Fertilization failure in IVF: why and what next?. Hum Reprod.

[B12] Westerlaken vdL, Helmerhorst F, Dieben S, Naaktgeboren N (2005). Intracytoplasmic sperm injection as a treatment for unexplained total fertilization failure or low fertilization after conventional in vitro fertilization. Fertil Steril.

